# Single and repeated moderate consumption of native or dealcoholized red wine show different effects on antioxidant parameters in blood and DNA strand breaks in peripheral leukocytes in healthy volunteers: a randomized controlled trial [ISRCTN68505294]

**DOI:** 10.1186/1475-2891-4-33

**Published:** 2005-11-14

**Authors:** Bianca M Arendt, Sabine Ellinger, Klaudia Kekic, Leonie Geus, Rolf Fimmers, Ulrich Spengler, Wolfgang-Ulrich Müller, Roland Goerlich

**Affiliations:** 1 Department of Hemostasis and Transfusion Medicine, School of Medicine, University of Duesseldorf, Moorenstr. 5, 40225 Duesseldorf, Germany; 2 Institute for Medical Biometry, Informatics and Epidemiology, University Hospital of Bonn, Sigmund-Freud-Str. 25, 53105 Bonn, Germany; 3 Department of General Internal Medicine, University Hospital of Bonn, Sigmund-Freud-Str. 25, 53105 Bonn, Germany; 4 Institute for Medical Radiobiology, University Duisburg-Essen, 45122 Essen, Germany; 5 Institute for Molecular Biotechnology, RWTH Aachen, Worringerweg 1, 52074 Aachen, Germany

## Abstract

**Background:**

Red wine (RW) is rich in antioxidant polyphenols that might protect from oxidative stress related diseases, such as cardiovascular disease and cancer. Antioxidant effects after single ingestion of RW or dealcoholized RW (DRW) have been observed in several studies, but results after regular consumption are contradictory. Thus, we examined if single or repeated consumption of moderate amounts of RW or DRW exert antioxidant activity *in vivo*.

**Methods:**

Total phenolic content and concentration of other antioxidants in plasma/serum, total antioxidant capacity (TEAC) in plasma as well as DNA strand breaks in peripheral leukocytes were measured in healthy non-smokers A) before, 90 and 360 min after ingestion of one glass of RW, DRW or water; B) before and after consumption of one glass of RW or DRW daily for 6 weeks. DNA strand breaks (SB) were determined by single cell gel electrophoresis (Comet Assay) in untreated cells and after induction of oxidative stress *ex vivo *with H_2_O_2 _(300 μM, 20 min).

**Results:**

Both RW and DRW transiently increased total phenolic content in plasma after single consumption, but only RW lead to a sustained increase if consumed regularly. Plasma antioxidant capacity was not affected by single or regular consumption of RW or DRW. Effects of RW and DRW on DNA SB were conflicting. DNA strand breaks in untreated cells increased after a single dose of RW and DRW, whereas H_2_O_2 _induced SB were reduced after DRW. In contrast, regular RW consumption reduced SB in untreated cells but did not affect H_2_O_2 _induced SB.

**Conclusion:**

The results suggest that consumption of both RW and DRW leads to an accumulation of phenolic compounds in plasma without increasing plasma antioxidant capacity. Red wine and DRW seem to affect the occurrence of DNA strand breaks, but this cannot be referred to antioxidant effects.

## Background

Polyphenols show antioxidant activity *in vitro *and probably also *in vivo *and therefore might protect from oxidative stress related diseases such as cardiovascular disease, cancer, or chronic inflammatory processes [1,2].

Red wine is rich in polyphenols [3,4] and is one of the main polyphenol sources in western countries [5,6]. Acute antioxidant effects after single ingestion of native and dealcoholized red wine as measured by increased antioxidant capacity in plasma or serum [7-12], reduced susceptibility of LDL to oxidation *ex vivo *[9], decreased plasma and urinary of F_2_-isoprostane concentrations [13] or protection from radiation-induced DNA damage [14] have been shown in several studies. Likewise, regular intake of red wine, dealcoholized red wine or red wine extract for 10 – 30 days induced antioxidant effects in some studies [15-20], but not in all [21-23]. Results of Cartron *et al. *[24] and van Golde *et al. *[25] even suggest prooxidant effects if red wine is consumed regularly. Hence it remains unclear if regular consumption of native or dealcoholized red wine can increase plasma antioxidant capacity and protects from oxidation *in vivo*. However, a sustained antioxidant response is necessary to achieve protection from oxidative stress related diseases. Hence, the aim of our study was to investigate if regular intake of moderate amounts of red wine could improve antioxidant parameters in healthy subjects, and if dealcoholized red wine could be an alternative source providing polyphenols without increasing the alcohol intake. We conducted two randomized, controlled intervention trials to examine the effects of single and repeated intake of native and dealcoholized red wine, respectively, on total phenolic content and antioxidant parameters in blood as well as on DNA strand breaks in peripheral leukocytes in healthy volunteers.

## Methods

### Subjects

Men and women, aged 18 – 50 y, free from any known diseases were included in the study. Smoking, excessive exercise (> 10 h/wk), pregnancy and lactation, present or former alcohol or drug abuse, medications interfering with antioxidants or intake of alcohol, supplementation of vitamins, minerals or probiotics (2 weeks before or during the study) were exclusion criteria. The study was performed according to the guidelines of the Declaration of Helsinki and was approved by the ethics committee of the University of Bonn. All subjects gave their informed written consent.

### Study design

The study consisted of two parts, a single-dose analysis investigating the effects of a single ingestion of red wine (RW) versus dealcoholized red wine (DRW) and a long-term dietary intervention trial in which RW and DRW were consumed daily for 6 wk. Both trials were prospective, randomized and controlled studies.

### Single-dose analysis

After an overnight fast, 27 healthy non-smokers (demographic data are shown in Table 1) were randomized to receive a single dose of 200 mL red wine (group RW; n = 9), 175 mL dealcoholized red wine (group DRW; n = 9), or 200 mL mineral water (controls; n = 9). Total phenolic content, total antioxidant capacity and concentrations of the major antioxidants (ascorbic acid, uric acid, albumin, bilirubin) in plasma, as well as DNA strand breaks in leukocytes (with and without *ex vivo *H_2_O_2 _treatment to induce oxidative stress) were measured before, 90 and 360 min after ingestion. Subjects were instructed to abstain from polyphenol rich foodstuff and from any alcoholic drinks 24 h before the first blood sampling until completion of the study. For this purpose they received a list of acceptable foods which are considered to be low in polyphenols. Compliance was controlled by a self-estimated 24-h dietary record. One hour after the first blood sampling, participants could choose from a breakfast buffet composed of foods low in polyphenols (white bread, butter, curd cheese, yogurt, cheese, honey, milk, fruit infusion, mineral water). All subjects were allowed to leave the study center at 90 min, when the initial blood samples had been drawn, and returned at 360 min for the follow-up blood samples. They were allowed to have lunch in between following the dietary restrictions concerning antioxidants.

**Table 1:** Characteristics of the subjects of the single-dose and the dietary intervention trial

	Single-dose analysis			Dietary intervention trial		
Group	RW	DRW	Controls	RW	DRW	Controls

Subjects (f/m), *n*	9 (7/2)	9 (8/1)	9 (6/3)	24 (12/12)	25 (15/10)	25 (15/10)
Age, *years*	27.1 ± 9.0^a^	26.2 ± 3.5^b^	31.4 ± 5.8^a,b^	30.0 ± 8.3	26.7 ± 6.4	28.8 ± 7.1
BMI *kg/m^2^*	20.8 ± 1.1	21.8 ± 1.9	22.0 ± 2.8	23.5 ± 3.3^c,d^	21.9 ± 2.1^c^	21.4 ± 2.2^d^
Exercise *h/week*	3.4 ± 2.9	2.7 ± 3.3	1.9 ± 3.3	2.2 ± 2.2	2.0 ± 2.0	2.9 ± 2.4

RW: Red wine; DRW: dealcoholized red wine; f: female, m: male

Values given are means ± SD. Values marked with identical letters differ, *p* ≤ 0.05 by Mann-Whitney U-test

### Dietary intervention trial

Seventy-eight healthy subjects were enrolled in the dietary intervention trial. They ingested 200 mL of red wine (group RW, 27 subjects) or 175 mL dealcoholized red wine (group DRW, 26 subjects) daily within one hour after dinner for 6 wk. The control group (25 subjects) did not receive any study drink. Subjects were instructed not to drink more than 2 cups (150 mL each) of coffee, black or green tea, and 2 glasses (200 mL each) of fruit juice per day, and to renounce from grape juice, multivitamin juices, and alcoholic beverages starting one week before the intervention throughout the whole study period.

Blood samples were drawn before and after intervention after an overnight fast (between 07.30 and 09.00 a.m.), and about 12 h after the last ingestion of RW or DRW. In addition to the laboratory parameters measured in the single-dose analysis, α-tocopherol concentration in serum was determined as changes are expected only in the long term [26]. To control compliance to dietary restrictions and assess possible changes of dietary patterns due to seasonal variations during the study period, self-estimated 7-day dietary records had to be completed in the week before and in the last week of intervention.

### Study drinks

The red wine used in the present studies was Spätburgunder, 1999, Marienthaler Klostergarten, Ahr, Germany. Dealcoholized red wine was produced by vacuum extraction of alcohol from the same batch. The amounts of flavonoids and phenolic acids ingested from a single serving of RW (200 mL) and DRW (175 mL) are listed in Table 2. The application of 175 mL DRW based on the assumption that 12.5% of the volume (25 mL / 200 mL) would be lost due to alcohol extraction, which would increase polyphenol concentration in DRW. However, subsequent analysis revealed a lower polyphenol content in DRW due to the processing. Thus, intake of polyphenols, especially flavonoids, was slightly lower from 175 mL DRW compared to 200 mL RW. The water for the control group in the single-dose analysis was Markus Brunnen "Still" (Vereinte Mineral- und Heilquellen, Rosbach, Germany), a carbonated natural mineral water from which iron is removed.

**Table 2:** Polyphenol intake from a single serving of red wine or dealcoholized red wine

	RW	DRW
Serving, *mL*	200	175
Total phenolics,^3 ^*mg CE*	293.2	271.6
TEAC, *mmol/L*	3.8	2.7
Phenolic acids		
Gallic acid, *mg*	8.0	9.4
Caffeic acid,^1 ^*mg*	3.7	3.1
p-Coumaric acid,^2 ^*mg*	0.7	0.8
Flavonoids		
Catechin, *mg*	26.5	10.8
Epicatechin, *mg*	14.4	8.5
Malvidin, *mg*	8.5	4.7
Peonidin, *mg*	1.0	0.5

RW: Red wine; DRW: dealcoholized red wine; CE: catechin equivalents; TEAC: trolox equivalent antioxidant capacity

^1^calculated from caftaric acid

^2^calculated from p-coumaroyl-glucosyl-tartrate

^3^Folin method

### Dietary intake of polyphenols

The subjects received a standardized dietary record which they completed for 24 h (single-dose analysis) or 7 days (dietary intervention trial), respectively. To determine polyphenol intake as exactly as possible, polyphenol rich foods were listed in detail. Calculation of the intake of flavonoids (kaempferol, quercetin, myricetin, catechin, epicatechin, epigallocatechin, gallocatechin, naringenin, cyanidin, peonidin, petunidin, and malvidin) and phenolic acids (salicylic, p-hydroxy benzoic, gallic, syringic, and ellagic acid) was based on data of Linseisen *et al. *[27] and Radtke *et al. *[28], which were completed by data for quercetin and kaempferol in tomato products [29] and catechin and epicatechin in apples, red grapes [30], and black tea [31].

### Collection of samples

Peripheral venous blood was collected in Vacutainer^® ^tubes (Becton Dickinson, Heidelberg, Germany) containing Li-heparin or no anticoagulant. Samples were protected from light and stored on ice until centrifugation (3000 × g, 20 min, 4°C). Plasma samples for determination of total phenolic content and antioxidant capacity were stored at -70°C, and for determination of albumin, uric acid and bilirubin at -20°C. For measurement of ascorbic acid, plasma was mixed with 5% trichloro acetic acid and centrifuged (3 min, 12000 × g). Supernatants were stored at -70°C. Serum was frozen at -20°C until measurement of α-tocopherol. For determination of DNA damage, heparinized blood was kept in the dark at room temperature until processing 60 – 120 min after sampling.

### Antioxidants in plasma and serum

Total phenolic content in plasma (TPP) was determined by the Folin-Ciocalteu method modified by Serafini *et al. *[10] to avoid plasma protein interference. Unlike Serafini *et al. *[10], we centrifuged the thawed plasma samples at 12000 × g for 5 min. To remove plasma proteins completely, 2 mol/L metaphosphoric acid (Merck, Darmstadt, Germany) was used for precipitation and an additional centrifugation step (2700 × g, 3 min) was introduced for the combined supernatants before adding Folin-Ciocalteu reagent (Fluka Chemie, Buchs, Switzerland). Experiments were performed in duplicate.

Plasma antioxidant capacity was determined by the Trolox equivalent antioxidant capacity (TEAC) assay as described previously [32]. The antioxidant capacity is given in comparison to a 1 mmol/L standard solution of 6-hydroxy-2,5,7,8-tetramethylchroman-2-carboxylic acid (Trolox) (Sigma, Deisenhofen, Germany). Experiments were performed in triplicate.

To control, if antioxidants other than polyphenols could have an impact on TEAC, the following major antioxidants in blood were also measured. Vitamin C concentration in plasma was determined colorimetrically according to Speitling *et al. *[33]. Experiments were performed in duplicate. Uric acid, albumin and bilirubin in plasma were determined by routine procedures at the Institute for Clinical Chemistry and Laboratory Medicine, University Duesseldorf, Germany. Concentration of α-tocopherol in serum was measured by High Performance Liquid Chromatography at the Research Centre for Diabetes, Duesseldorf, Germany.

### DNA strand breaks in peripheral leukocytes

Single Cell Gelelectrophoresis (Comet Assay) was performed to assess DNA strand breaks in peripheral leukocytes. In this assay, cells are embedded in agarose on slides, incubated in PBS (control cells) or H_2_O_2 _(induction of oxidative stress), and lysed. During subsequent electrophoresis, DNA regions that are relaxed due to strand breaks migrate towards the anode forming a typical comet structure.

This assay was performed according to the standard protocol of Bauch *et al. *[34] adapted to the use of hydrogen peroxide as a stressor. To simulate the *in vivo *situation as far as possible and to avoid artifacts by density centrifugation, whole blood was used instead of isolated mononuclear cells. Ten μL heparinized whole blood were diluted with 90 μL PBS (10 mmol/L, pH 7.4, without Ca^2+^/Mg^2+^) (Sigma) to obtain a single cell suspension. After preparation of the slides, they were incubated for 20 min at 4°C with either 300 μmol/L H_2_O_2 _(Sigma) in PBS to induce DNA strand breaks or in pure PBS (controls). Electrophoresis was performed at 4°C and 1 V/cm for 4 min on a flat-bed electrophoresis apparatus with electrode spacing of 39 cm (Mega Horizontal Gelbox Safety; Molecular Bio-Products, San Diego, USA). Experiments were performed in duplicate.

For analysis, gels were rehydrated in *aqua bidest*. (10 min, room temperature) and DNA was dyed with 150 μL propidium iodide (Sigma) (25 μmol/L, solved in 125 mmol/L Tris with 123 mmol/L NaCl in *aqua bidest*., pH 7.5). Measurements were performed with the image analysis software package Comet Assay II (Perceptive Instruments, Suffolk, UK) coupled to a fluorescence microscope (Leitz DM RB, Leica, Bensheim, Germany) with a CCD camera (Cohu, San Diego, USA). Fifty cells were measured on each slide and Tail Intensity (TI) and Tail Moment (TM) were calculated to quantify DNA strand breaks.

DNA strand breaks in untreated cells reflect the steady state between DNA damage and repair *in vivo *[35-37] and are therefore referred to as "endogenous" DNA damage in this paper. "Exogenous" DNA strand breaks are those induced by treatment with H_2_O_2 _*ex vivo *and were calculated as the difference between treated and untreated cells.

### Sample size calculation

The sample size calculation was based on plasma antioxidant capacity (TEAC), which was considered as main outcome and based on data from a previous study with healthy subjects who ingested polyphenol rich fruit-juices or a fruit-vegetable concentrate [32]. With 6 subjects in each group (RW, ERW, controls) the single-dose analysis has a power of 90% to detect TEAC changes >0.033 mmol/L, which was the mean difference between two measurements in the previous study. To account for possible drop-outs, we included 9 subjects in each group, adding up to a total of 27 participants.

Mean TEAC difference in the previous long-term study was 0.075 mmol/L. To detect changes ≥ 0.075 mmol/L and differences between the groups with a power of 90%, a sample size of 25 subjects in each group (RW, DRW, controls) was calculated. Level of significance was 5% for all tests.

### Statistical analysis

Values given are means ± SD unless indicated otherwise. Differences between the groups were examined using the Mann-Whitney U-test. The effects of the study drinks on the investigated parameters were evaluated by comparing the values obtained at different time intervals with baseline for each group using the Wilcoxon signed rank test. Differences indicated by *p *< 0.05 were considered statistically significant. Statistical tests were performed with SPSS 10.0 for Windows (SPSS Inc., Chicago, IL, USA).

## Results

### Single-dose analysis

The day before the study the subjects ingested 22.9 ± 47.4 mg polyphenols (Flavonoids: 2.1 ± 5.3 mg; Phenolic acids: 20.8 ± 46.2 mg). This corresponds to 8 – 16% of the average intake observed in another German cohort [27,28] and in our dietary intervention trial (see below) indicating good compliance to the dietary restrictions.

Results for the antioxidant parameters in plasma are presented in Table 3. Total phenolic content in plasma increased 90 min after ingestion of RW (*p *= 0.008) or DRW (*p *= 0.008). After 360 min values were higher than at baseline in groups RW (*p *= 0.02) and DRW (*p *= 0.008), but also in control subjects (*p *= 0.01). Vitamin C concentration in plasma decreased 360 min after RW consumption (*p *= 0.008), but increased in group DRW (*p *= 0.02). Significant changes of endogenous antioxidants, i.e. uric acid, albumin or bilirubin, have been observed after consumption of RW and DRW, and also in the control group, but TEAC was not altered significantly in any group.

**Table 3:** Antioxidant parameters in healthy volunteers after single ingestion of native or dealcoholized red wine

	RW	DRW	Controls
Parameter			
TPP, *mg CE/L*			
Baseline	15.1 ± 2.0	15.9 ± 2.0	13.9 ± 2.8
90 min	17.7 ± 1.8*^a^	18.3 ± 2.5* ^b^	14.3 ± 3.5^a,b^
360 min	15.5 ± 1.8*	16.6 ± 2.0*	14.3 ± 2.7*
TEAC, *mmol/L*			
Baseline	1.22 ± 0.08	1.25 ± 0.08	1.23 ± 0.05
90 min	1.25 ± 0.07	1.28 ± 0.08	1.26 ± 0.05
360 min	1.23 ± 0.08	1.24 ± 0.07	1.24 ± 0.04
Vitamin C, *mg/dL*			
Baseline	1.33 ± 0.26	1.15 ± 0.18	1.14 ± 0.30
90 min	1.27 ± 0.21	1.18 ± 0.18	1.17 ± 0.35
360 min	1.11 ± 0.22*	1.35 ± 0.32*	1.14 ± 0.26
Uric acid, *mg/dL*			
Baseline	4.6 ± 1.0	4.8 ± 1.2	4.6 ± 1.1
90 min	5.3 ± 1.0*	4.9 ± 1.1	4.9 ± 1.2
360 min	4.5 ± 0.9	4.5 ± 1.0	4.4 ± 1.1
Albumin, *g/dL*			
Baseline	4.1 ± 0.4	4.4 ± 0.4	4.1 ± 0.4
90 min	4.4 ± 0.4	4.4 ± 0.5	4.3 ± 0.3*
360 min	4.4 ± 0.4	4.4 ± 0.5	4.4 ± 0.5
Bilirubin, *mg/dL*			
Baseline	0.73 ± 0.44	0.69 ± 0.27	0.54 ± 0.23
90 min	0.71 ± 0.37	0.68 ± 0.22	0.61 ± 0.27*
360 min	0.52 ± 0.29*	0.53 ± 0.26*	0.50 ± 0.22
TM_0_, *arbitrary units*			
Baseline	1,86 ± 0,48	1,98 ± 0,33	2,19 ± 0,67
90 min	2,03 ± 0,43	2,36 ± 0,23	2,43 ± 0,43
360 min	2,61 ± 0,43*	2,67 ± 0,24*	2,33 ± 0,44
TM_300_, *arbitrary units*			
Baseline	1,69 ± 0,92	1,43 ± 0,78	1,15 ± 1,07
90 min	1,39 ± 0,65	1,05 ± 0,87	0,78 ± 1,23
360 min	1,22 ± 0,67^c^	0,21 ± 0,45*^c,d^	1,21 ± 0,52^d^

Amounts of study drinks ingested were 200 mL red wine (RW), 175 mL dealcoholized red wine (DRW) or 200 mL water (Controls)

TPP: total phenolic content in plasma; CE: catechin equivalents; TEAC: trolox equivalent antioxidant capacity; TM_0_: Tail Moment in untreated cells (endogenous DNA strand breaks);

TM_300_: Tail Moment in cells treated with 300 μM H_2_O_2 _for 20 min (exogenous DNA strand breaks)

Values are means ± SD, n = 9 for all experiments except for control group at 90 min, n = 7 for all parameters; DRW, n = 8 for TM_0_, TM_300_, and control group at 360 min, n = 6 for TM_0_, TM_300_

*Values different from baseline, P < 0.05 by Wilcoxon signed rank test

^a-d^Values marked with identical letters differ between groups, P < 0.05 by Mann-Whitney U-test

**Figure 1:**
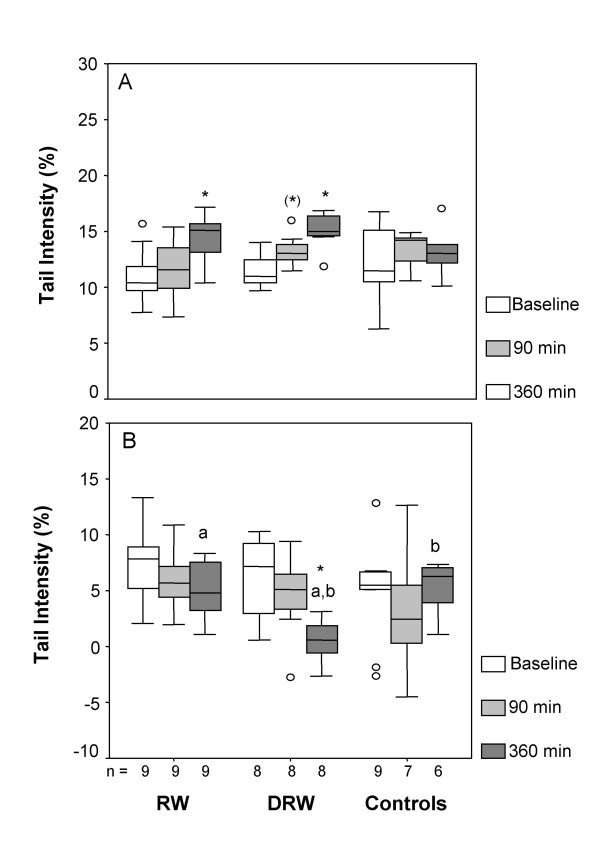
**DNA strand breaks in peripheral leukocytes after single ingestion of native or dealcoholized red wine**. A) Tail Intensity in untreated cells (endogenous DNA strand breaks) B) Tail Intensity in cells treated with 300 μM H_2_O_2 _for 20 min (exogenous DNA strand breaks)Amounts of study drinks ingested were 200 mL red wine (RW), 175 mL dealcoholized red wine (DRW) or 200 mL water (Controls) The box represents the distribution falling between the 25^th ^and 75^th ^percentiles, with the median as the horizontal line within the box. The whiskers connect the largest and smallest values not categorized as outliers or extreme values, which are represented by single data points. *, ** Values different from baseline, *P < 0.05; ** P < 0.01 by Wilcoxon signed rank test ^a,b^Values marked with identical letters differ between groups, P < 0.01 by Mann-Whitney U-test

Endogenous DNA strand breaks increased 360 min after consumption of either RW or DRW compared to baseline values. In group DRW an increase could be observed after 90 min, but only with borderline significance (*p *= 0.05). Hydrogen peroxide-induced DNA strand breaks decreased in group DRW at 360 min, whereas no alterations could be observed after RW consumption or in controls. Statistical analysis provides the same results for TI (Figure 1) and TM (Table 3).

### Dietary intervention trial

Four subjects were lost during the study. One woman in group DRW did not tolerate the study drink. The other subjects were allocated to group RW. Of those, one woman fell ill, which was not related to the intervention. One man started smoking during the study and the other one was lost to follow-up. Demographic data of the 74 subjects who completed the study are summarized in Table 1.

Flavonoid intake increased significantly in group RW (28.9 ± 16.6 vs. 76.7 ± 24.5 mg/d; *p *< 0.001) and DRW (39.1 ± 20.5 vs. 64.2 ± 27.6 mg/d; *p *< 0.001), but not in controls (37.7 ± 21.3 vs. 41.0 ± 24.8 mg/d). During intervention, the flavonoid intake in group RW exceeded that in group DRW (*p *= 0.005), and it was higher in group RW (*p *< 0.001) and DRW (*p *= 0.005) than in controls. Intake of phenolic acids increased during supplementation of RW (107.8 ± 75.5 vs. 135.1 ± 82.5 mg/d; *p *= 0.01) and DRW (123.5 ± 83.2 vs. 158.3 ± 102.7 mg/d; *p *= 0.02), but also in controls (87.2 ± 69.7 vs. 135.6 ± 100.3 mg/d; *p *= 0.03). The amounts did not differ between the groups before or during intervention.

The difference to the average intake of 54 mg/d flavonoids [27] and 222 mg/d phenolic acids [28] reported by others could be explained by the restriction of polyphenol rich foods, especially coffee, which is a major source of phenolic acids in Germany [28].

Total phenolic content in plasma (Table 4) increased after 6 wk regular consumption of RW (*p *= 0.002), but did not change in group DRW or controls. A significant increase of plasma uric acid concentration was observed in controls (*p *= 0.04), and a decrease of bilirubin in group DRW (*p *= 0.02) (Table 4). In contrast, vitamin C and albumin in plasma, α-tocopherol in serum, and plasma antioxidant capacity did not change significantly in any group (Table 4). There was no significant difference between the groups before and after 6 wk of intervention considering the antioxidant parameters.

**Table 4:** Antioxidant parameters in healthy volunteers after regular consumption of native or dealcoholized red wine

	RW	DRW	Controls
Subjects, *n*	24	25	25

Parameter			
TPP, *mg CE/L*			
Baseline	15.6 ± 1.6	15.6 ± 2.1	15.5 ± 1.4
6 wk	16.4 ± 1.4*	16.0 ± 2.0	15.3 ± 2.2
TEAC, *mmol/L*			
Baseline	1.47 ± 0.06	1.44 ± 0.08	1.46 ± 0.08
6 wk	1.45 ± 0.04	1.42 ± 0.07	1.43 ± 0.07
Vitamin C, *mg/dL*			
Baseline	1.30 ± 0.28	1.43 ± 0.36	1.48 ± 0.29
6 wk	1.35 ± 0.26	1.42 ± 0.39	1.57 ± 0.28
α-Tocopherol, *mg/dL*			
Baseline	11.3 ± 3.0	11.2 ± 2.6	11.2 ± 2.9
6 wk	11.3 ± 3.0	11.5 ± 2.7	11.0 ± 2.7
Uric acid, *mg/dL*			
Baseline	4.7 ± 1.2	4.4 ± 1.1	4.4 ± 1.2
6 wk	4.9 ± 1.2	4.5 ± 0.9	4.7 ± 1.5*
Albumin, *g/dL*			
Baseline	4.2 ± 0.4	4.3 ± 0.4	4.2 ± 0.4
6 wk	4.3 ± 0.4	4.1 ± 0.6	4.2 ± 0.5
Bilirubin, *mg/dL*			
Baseline	0.68 ± 0.28	0.68 ± 0.33	0.64 ± 0.33
6 wk	0.61 ± 0.35	0.59 ± 0.27*	0.62 ± 0.35
TM_0_, *arbitrary units*			
Baseline	2,18 ± 0,56	1,97 ± 0,56	2,22 ± 0,79
6 wk	1,88 ± 0,48*	2,05 ± 0,70	1,95 ± 0,49
TM_300_, *arbitrary units*			
Baseline	1,02 ± 0,69	0,87 ± 0,72	0,95 ± 0,54
6 wk	0,96 ± 0,49	0,92 ± 0,65	1,18 ± 0,50

Amounts ingested daily for 6 weeks were 200 mL red wine (RW) or 175 mL dealcoholized red wine (DRW). Control subjects did not receive any study drink.

TPP: total phenolic content in plasma; CE: catechin equivalents; TEAC: trolox equivalent antioxidant capacity; TM_0_: Tail Moment in untreated cells (endogenous DNA strand breaks);

TM_300_: Tail Moment in cells treated with 300 μM H_2_O_2 _for 20 min (exogenous DNA strand breaks)

Values are means ± SD

*Values different from baseline, P < 0.05 by Wilcoxon signed rank test

**Figure 2:**
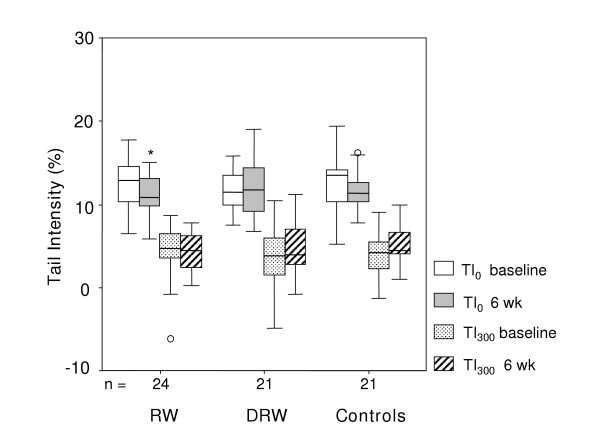
**DNA strand breaks in peripheral leukocytes after regular consumption of native or dealcoholized red wine**. Amounts ingested daily for 6 weeks were 200 mL red wine (RW) or 175 mL dealcoholized red wine (DRW). Control subjects did not receive any study drink. TI_0_: Tail Intensity in untreated cells (endogenous DNA strand breaks); TI_300_: Tail Intensity in cells treated with 300 μM H_2_O_2 _for 20 min (exogenous DNA strand breaks)Boxes represent the distribution falling between the 25^th ^and 75^th ^percentiles, with the median as the horizontal line within the box. The whiskers connect the largest and smallest values not categorized as outliers or extreme values, which are represented by single data points. *Value different from baseline, P < 0.05 by Wilcoxon signed rank test.

Endogenous DNA strand breaks (Figure 2, Table 4) decreased only after regular consumption of RW (TI: *p *= 0.03; TM: *p *= 0.04), but not in group DRW and controls. Susceptibility of leukocytes towards H_2_O_2_-induced DNA damage was not altered in any group. Differences in endogenous or *ex vivo *induced DNA strand breaks could not be observed between the groups, neither before nor after 6 wk intervention (Figure 2, Table 4).

## Discussion

The present studies demonstrate that polyphenols from RW and DRW are bioavailable and accumulate in plasma after regular consumption. Even though total antioxidant capacity in plasma did not change after RW or DRW consumption, both drinks affected DNA strand breaks in peripheral leukocytes. Results are contradictory, as DNA damaging effects of RW and DRW occur after single ingestion, whereas regular RW consumption seems to protect from endogenous DNA strand breaks. Further investigations are required to clarify whether interactions of red wine polyphenols with cellular DNA result in genotoxic or anticarcinogenic effects.

The results of the single-dose analysis confirm that polyphenols are bioavailable from both RW and DRW [8,10,11], and that alcohol does not significantly affect polyphenol absorption in humans [38-40]. Accumulation of phenolic compounds in plasma could be measured after regular consumption of RW corresponding to previous findings [16,19] but not in group DRW. We assume that this is rather due to the slightly lower intake of polyphenols from DRW than to different alcohol content [38-40].

The impact of phenolic compounds from RW and DRW on the pro-/antioxidant balance in plasma seems to be low, as plasma antioxidant capacity was neither altered significantly in the short nor in the long term as also reported by others [7,20,22,23]. There was no effect of RW or DRW consumption on antioxidant vitamins in the dietary intervention trial corresponding to the findings of previous studies [17,20]. Interestingly, the concentration of vitamin C in plasma increased 360 min after consumption of one single dose of DRW whereas it decreased after RW suggesting that the polyphenols might act as antioxidants *in vivo *but this is counteracted by prooxidant effects of alcohol in native RW [13,23]. Changes in vitamin C levels did not have a significant impact on plasma antioxidant capacity but might have protected cellular DNA from oxidation *ex vivo*, which will be discussed later on.

Uric acid, one of the main antioxidants contributing to plasma antioxidant capacity [13,32], increased after single RW consumption as expected [13,41]. However, the increase of 15% in TEAC observed after 90 min, even in combination with a 17% increase of total phenolic content did not lead to a significant rise of plasma TEAC. This supports the observations of Cao *et al. *[7] implying that the TEAC method is not sensitive enough to measure small changes in the pro-antioxidant balance after ingestion of foods rich in polyphenols.

Further changes of albumin, uric acid and bilirubin concentrations occurred in both trials, probably due to normal biological variance, but they were not associated with changes of plasma TEAC.

In contrast to antioxidant capacity, there was a significant effect of RW and DRW on DNA strand breaks in peripheral leukocytes suggesting interactions of red wine polyphenols with cellular DNA. Regular consumption of RW protects cellular DNA from strand breaks *in vivo*, which corresponds to findings of previous studies [18,42], probably due to DNA stabilizing and/or antioxidant effects of RW polyphenols [43]. In contrast, single ingestion of RW or DRW increased endogenous DNA strand breaks. This was not expected as DNA damaging effects of RW polyphenols have been observed only *in vitro *and for concentrations of 25–1000 μM [44-46], whereas in human plasma polyphenols occur mainly as metabolites and in concentrations below 1 μM [6]. As the same outcome was found in groups RW and DRW, genotoxic effects of alcohol [47] can also be excluded. Since strand breaks occur during DNA repair via base or nucleotide excision [35], increased DNA strand breaks as observed in our study might also result from activation of DNA repair enzymes by polyphenols [48]. To clarify the mechanisms, a modified protocol for the single cell gel electrophoresis allowing to separately assessing oxidation of purine and pyrimidin bases [35] as well as DNA repair kinetics [49] should be used in future studies.

The discrepancy between increased DNA strand breaks after single and reduced DNA strand breaks after repeated consumption of RW might be due to different polyphenols which might have been present in plasma or leukocytes after single or regular consumption, respectively. This would be plausible regarding the different elimination half lives of the various RW polyphenols and their metabolites [6,39,50,51], but quantitative analysis of single polyphenolic compounds in plasma/cells would be necessary to confirm this. Furthermore, the different mode of RW consumption in the short- and the long-term study (after an overnight fast vs. regularly after dinner) could also provide an explanation. Since consumption of RW with meals reduces prooxidative effects occurring post-prandially [24,52,53], the DNA protective effects of RW in our dietary intervention trial might have resulted from reduced prooxidative effects of the meals, which were lacking in the single-dose analysis.

Exogenous DNA strand breaks, which reflect the activity of non-enzymatic antioxidants [35,37], have only been reduced after single consumption of DRW, which corresponds to the results of a recent study showing that consumption of 300 mL DRW protected lymphocytes from radiation-induced DNA damage *ex vivo *[14]. As in our study the plasma phenolic content was not different between groups RW and DRW, the increased vitaminC concentration in plasma observed in group DRW might have protected leukocyte DNA against oxidative stress *ex vivo*. Indeed, DNA protecting effects of vitamin C have been described *in vitro *[54,55] and are also supposed to occur *in vivo *[36,56,57]. This is also supported by Greenrod *et al. *[14] who observed that the contribution of catechin to DNA protecting effects or DRW is quite small, and that other factors seem to play an important role.

It has to be noted that in the dietary intervention trial consumption of RW and DRW lead to an increase in flavonoid intake compared to baseline and to controls, whereas phenolic acid consumption increased in RW, DRW and controls after 6 weeks compared to baseline without any differences between the groups. This is probably due to seasonal variations of the food pattern as our study period was between May and July, when consumption of fresh fruit rich in phenolic acids increased (data not shown). Hence, the observed effects of RW on DNA strand breaks in peripheral leukocytes can be attributed to the additional intake of flavonoids rather than to phenolic acids.

The major advantage of our study compared to others is the investigation of both single and regular consumption of RW and DRW which has only been done so far by Cartron *et al. *[24]. In both studies substantial differences between short- and long-term effects occurred implying that single-dose analysis alone are not appropriate to investigate potential health effects of red wine consumption.

However, our study has some constraints, regarding the different polyphenol intake with RW and DRW, which does not allow a direct comparison of both study drinks. In future studies, drinks with equal polyphenol composition should be applied. Moreover, in the single dose analysis TPP increased 360 min after intervention in groups RW and DRW, but also in controls. This could be due to 1) unknown amounts of polyphenols in "permitted" foods (e.g. white bread or pasta). These foods are generally considered to be low in polyphenols and thus, are not listed in the polyphenol database [27,28]. Nevertheless, they may have provided considerable amounts of polyphenols, especially phenolic acids [50] leading to an increase of TPP in the control group. 2) As food intake at lunchtime was not recorded, we cannot exclude that the volunteers failed to comply with the polyphenol poor diet in the time interval between blood sampling at 90 and 360 min. To avoid these confounding factors in future short-term studies we strongly recommend to provide the subjects with standardized amounts of polyphenol free formula diets instead of foods low in polyphenols ad libitum.

It is unlikely that random inhomogeneities of the groups (Table 1) would have influenced our results. In the single-dose analysis, control subjects were only slightly older (≈ 5 years) than those in group RW and DRW and therefore metabolic alterations are not expected. In the dietary intervention trial, the higher body weight and BMI in group RW could have reduced the response to RW, as the intake of polyphenols / kg body weight was lower in that group. However, effects on plasma phenolic content and DNA strand breaks occurred only with RW, and even more pronounced effects might have been observed in subjects with a lower body weight.

## Conclusion

One glass of RW or DRW is sufficient to transiently increase concentration of phenolic compounds in plasma, but accumulation after regular consumption could only be shown for RW. Polyphenols from RW and DRW seem to affect DNA damage in leukocytes, but antioxidant effects probably play only a minor role. Endogenous DNA strand breaks increased after a single dose of RW or DRW but could be reduced after 6 wk daily consumption of RW. As DNA damage is involved in cancer initiation, the mechanisms and the physiological relevance of these findings are worth being considered in future studies.

## Abbreviations

Red wine (RW); dealcoholized red wine (DRW); total antioxidant capacity (TEAC); total phenolic content in plasma (TPP).

## Competing interests

The author(s) declare that they have no competing interests.

## Authors' contributions

BMA participated in study design and coordination, carried out the trolox equivalent antioxidant capacity assay and the single cell gel electrophoresis, did the statistical analysis and drafted the article. SE was involved in study design and realization and helped to draft the manuscript. KK measured total phenolic content and vitamin C concentration in plasma, and analysed the dietary records together with LG. RF did the sample size calculation and contributed to the statistical analysis. US supervised the clinical part of the study. W-UM supported the implementation, data analysis and interpretation of the single cell gel electrophoresis. RG had the initial idea and supervised the study. All authors read and approved the final manuscript.
